# An Overview of Recent Progress in Nanofiber Membranes for Oily Wastewater Treatment

**DOI:** 10.3390/nano12172919

**Published:** 2022-08-24

**Authors:** Rosalam Sarbatly, Chel-Ken Chiam

**Affiliations:** 1 Chemical Engineering, Faculty of Engineering, Universiti Malaysia Sabah, Jalan UMS, Kota Kinabalu 88400, Sabah, Malaysia; 2 Nanofiber and Membrane Research Laboratory, Faculty of Engineering, Universiti Malaysia Sabah, Jalan UMS, Kota Kinabalu 88400, Sabah, Malaysia; 3 Oil and Gas Engineering, Faculty of Engineering, Universiti Malaysia Sabah, Jalan UMS, Kota Kinabalu 88400, Sabah, Malaysia

**Keywords:** oily wastewater, nanofiber, nanomaterial, membrane, wettability modification, flux, separation efficiency

## Abstract

Oil separation from water becomes a challenging issue in industries, especially when large volumes of stable oil/water emulsion are discharged. The present short review offers an overview of the recent developments in the nanofiber membranes used in oily wastewater treatment. This review notes that nanofiber membranes can efficiently separate the free-floating oil, dispersed oil and emulsified oil droplets. The highly interconnected pore structure nanofiber membrane and its modified wettability can enhance the permeation flux and reduce the fouling. The nanofiber membrane is an efficient separator for liquid–liquid with different densities, which can act as a rejector of either oil or water and a coalescer of oil droplets. The present paper focuses on nanofiber membranes’ production techniques, nanofiber membranes’ modification for flux and separation efficiency improvement, and the future direction of research, especially for practical developments.

## 1. Introduction

Discharging large volumes of oily wastewater is unavoidable due to the rapid growth of industries, such as food and beverage, textile, cosmetic, metallurgical manufacturing and petroleum production. In addition, the frequent accidents of oil spillages and chemical leakages have worsened the environmental pollution. The oily wastewater generally exists in three main categories [1]: suspended and free-floating oil (>150 μm), dispersed and unstable oil/water emulsion (20–150 μm) and stable oil/water emulsion (<20 μm). The oil fractions in the wastewaters’ first and second categories are easier to remove by conventional physical separation techniques, e.g., gravity separation, skimming, floatation, burning, etc. At the same time, the stable oil/water emulsion is treated with chemical and biological techniques. However, the conventional physical treatment techniques suffer low separation efficiency, high cost and high energy consumption. The chemical treatment method generates secondary pollutants and is costly; the biological treatment requires vast space and the processes are sensitive to temperature and pH.

Membrane separation technologies have recently received significant attention in oily wastewater treatment [2,3]. The strengths of using membrane separation for the oily wastewater treatment include high flux, excellent oil removal, light weight, mechanical flexibility, compact design with small space requirement, low energy consumption and low cost. However, fouling is the major challenge, which causes the flux declination.

Nanofibrous membranes have obtained significant attention in membrane separation, starting ten years ago. The nanofibrous membrane is a thin film comprising nanofibers that overlap with each other in a completely random manner. The nanofibrous membrane is well known in separation applications because of the fibers’ fine diameters, making the membrane highly porous. Although the foulants block some pore channels, the highly interconnected pore structures in the nanofibrous membrane permit the liquids to flow through other alternative paths. The permeation flux in the nanofibrous structures has been three-times higher than that of the phase inversion membranes [4,5]. Thus, the nanofiber membrane serves as a better fouling resistance. This short review article describes the techniques that are commonly used to produce the nanofibers, the membrane modification techniques which have further improved the permeation flux and the separation efficiency of oil/water, and the remarks of future research directions. 

## 2. An Overview of Nanofiber Production Techniques

**Figure 1:**
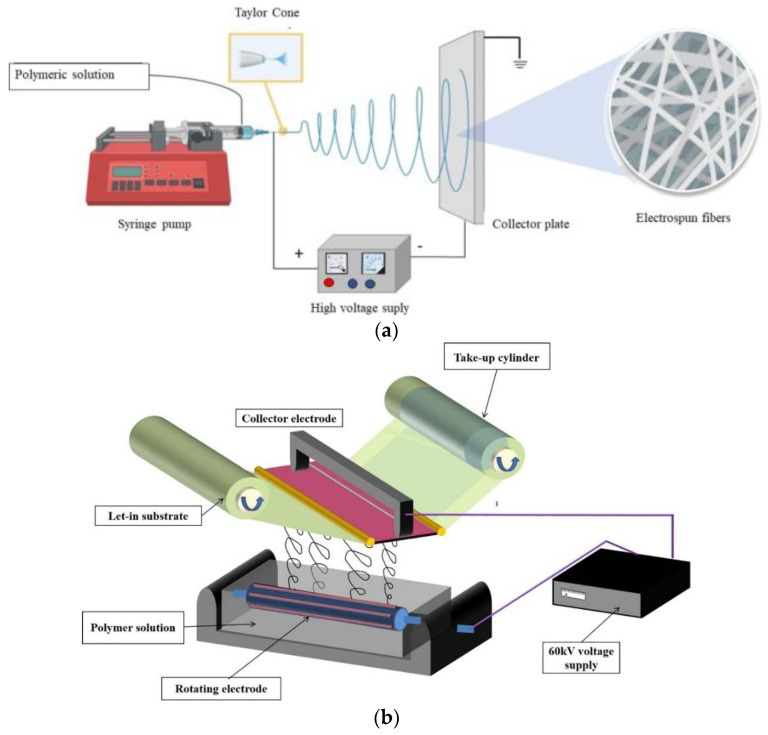

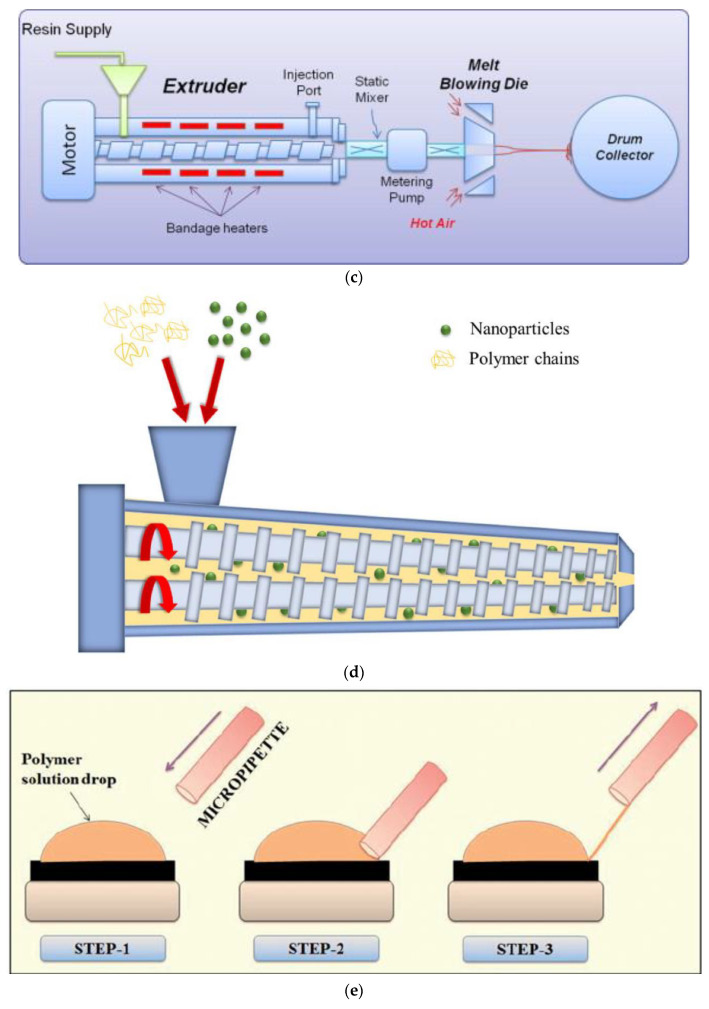

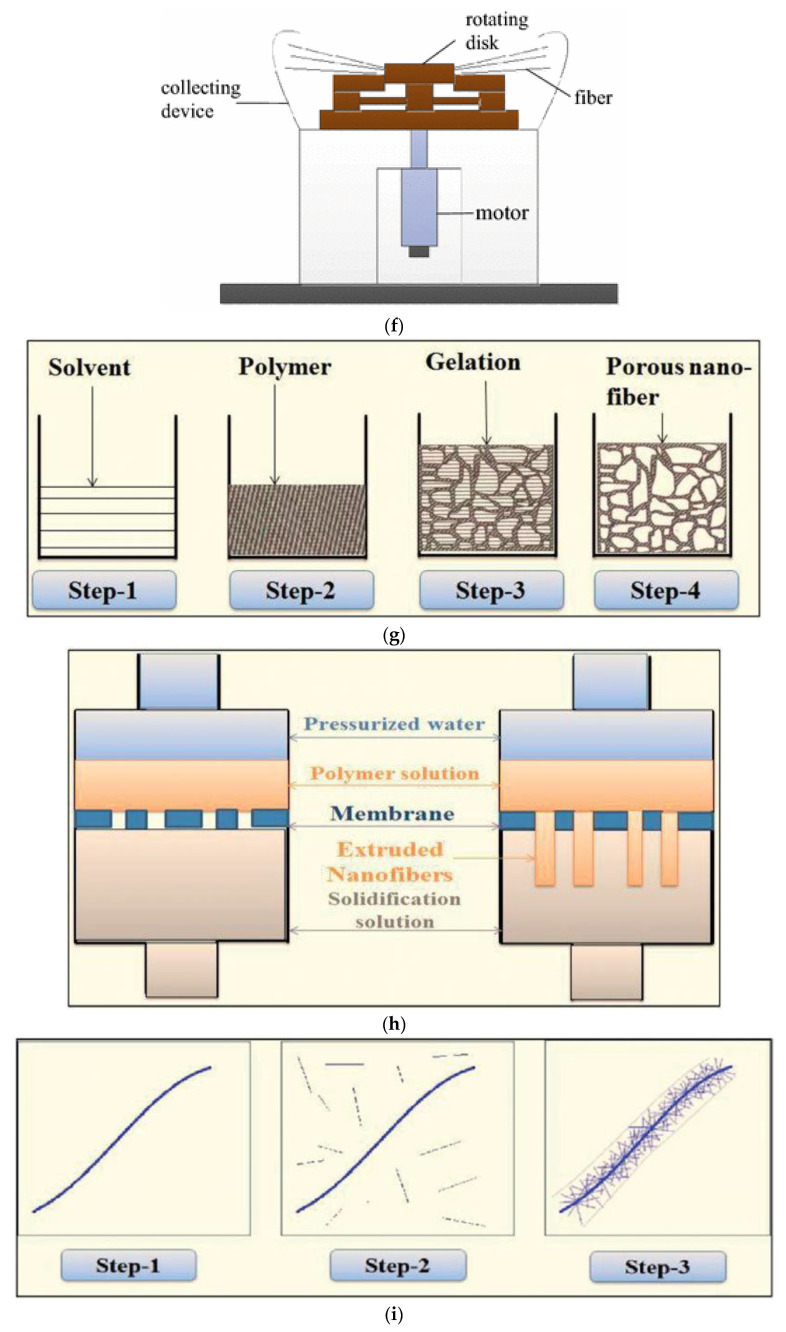
Various types of nanofiber production techniques. (**a**) Needle electrospinning, reproduced with permission from [21], copyright 2021 Springer Nature; (**b**) needleless electrospinning, reproduced with permission from [22], copyright 2021 Elsevier; (**c**) melt-blowing, reproduced with permission from [23], copyright 2013 Elsevier; (**d**) melt-blending, reproduced with [24], (**e**) drawing, reproduced with permission from [25], copyright 2014 Taylor & Francis; (**f**) centrifugal force spinning, reproduced with permission from [26], copyright 2018 Springer Nature; (**g**) phase separation, reproduced with permission from [25], copyright 2014 Taylor & Francis; (**h**) template synthesis, reproduced with permission from [25], copyright 2014 Taylor & Francis; and (**i**) self-assembly, reproduced with permission from [25], copyright 2014 Taylor & Francis.

Nanofiber membranes are popular in their wide range of applications in water separation and purification [6,7,8]. As shown in Figure 1, the nanofibers can be produced from various techniques, such as needle electrospinning [9], needleless electrospinning [10], melt-blowing [11], melt-blending extrusion [12], drawing [13], centrifugal force spinning [14], phase separation [15], template synthesis [16], self-assembly [17], etc. Table 1 shows the different techniques and the polymeric materials used to produce the nanofibers. A comparison of different nanofiber production techniques is summarized in Table 2. To date, the electrospinning technique is the most applied to produce nanofiber membranes for the oil/water separation [18], followed by melt-blowing [19] and melt-blending extrusion [20].

Electrospinning was first patented by Formhals in 1934 [72]. Electrospinning, also known as ‘electrostatic spinning’, is a versatile technique that applies electric force to produce fibers with diameters as small as hundreds of nanometers. The pressurized polymeric liquid exits from the syringe needle and is subjected to high-voltage DC power. The syringe needle is charged positively and the collector plate is negatively charged using a DC power supply. The collector plate is grounded. The nonwoven fibers are formed when the electrostatic repulsion curbs the surface tension of the polymeric liquid ejected from the syringe needle. The polymer–solvent evaporates during the electrospinning process. The diameters of the fibers that can be fabricated from the electrospinning process range from 3 nm to 5 μm [73,74,75] or greater [76,77]. The diameters of the fibers are basically controlled by the properties in the polymer solution, such as the polymer concentration [78,79], molecular weight of the polymer [80], conductivity [81] and solvent volatility [82]. The process parameters also significantly affect the diameter of the fibers, such as the spinning throughput and the applied voltage [83], temperature [79] and humidity [81,82].

The melt-blowing process to form fibers with diameters below 10 μm was first demonstrated by Van A. Wente in 1954 [84]. Melt-blowing is a one-step process, whereby the molten polymer emerges through an orifice of a die and is blown into fibers by hot and high-velocity air. The fibers are collected on a rotary drum. The melt-blowing process can produce nano- and micro-fibers with different operating settings. The average fiber diameter generally ranges from 2 to 4 μm; the minimum can range from 0.3 to 0.6 μm and the maximum is between 15 and 20 μm [85]. Hassan et al. [23] fabricated nanofiber melt-blown membranes from a metallocene isotactic polypropylene and the average fiber diameter ranged between 1 and 2 μm, with different die designs as a new strategy to produce the fiber size in a range of 300–500 nm. The melt-blowing process is a mass-producing fiber technique without using any polymer solvent, which can produce the fiber at rates between 500 and 1000 g/h. The melt-blowing process does not require massive-scale solvent recovery from the dilute air stream as the electrospinning method does.

Two polymers are blended and fed into the co-rotating twin-screw extruder in the melt-blending extrusion. The dispersed phase is stretched into nanofibers and the nanofiber membrane is obtained after removing the matrix phase. The diameters of the nanofibers range from 60 to 900 nm [20,53].

## 3. Parameters of Nanofiber Membrane Affecting the Oil/Water Separation Performance

In most laboratories, various binary oil/water systems are tested as the models of oily wastewater. The oil/water models are categorized into two types, which are the oil/water mixtures and the oil/water emulsions. Gravity-driven filtration [86] is the most straightforward testing process for oil/water separation. Some researchers have also used dead-end [87] and cross-flow filtration [88] methods to perform oil/water separation experiments. The water is recovered in the permeate stream and the oil is harvested in the rejection stream when a hydrophilic and oleophobic membrane is used, while a hydrophobic and oleophilic membrane rejects the water and permits the oil to permeate. The separation performance of the membrane is determined in terms of flux, separation efficiency, oil rejection and, sometimes, demulsification efficiency.

The flux (J) is measured as the volume of permeate produced per unit time per unit membrane area [86,87,88]:

(1) $$J=\frac{V}{At}$$

where V is the volume of the permeate, A is the membrane area and t is the duration time to collect the permeate. The flux is determined by the nanofiber membrane properties, such as pore size, porosity and fiber diameter. The flux increases with increasing the pore size, porosity and the number of interconnected pores due to the presence of more flow channels [89,90]. In addition, the nanofiber membrane exhibits good permeability and the flux increases when the nanofiber diameter increases [91] because the pore size and porosity of the nanofiber membrane increase correspondingly [91,92,93].

The separation efficiency (R) of the membrane is calculated as the total amount of the oil removed divided by the initial amount of the oil [94,95]:

(2) $$R\left(\%\right)=100\left(1-\frac{C_{p}}{C_{0}}\right)$$

where $C_{p}$ and $C_{0}$ are the oil contents in the permeate and feed, respectively, for the hydrophilic and oleophobic membrane used, i.e., water-removing mode. For oil-removing mode, $C_{p}$ and $C_{0}$ are the water contents in the permeate and feed, respectively, when the hydrophobic and oleophilic membrane is employed. Equation (2) is also known as oil rejection when water-removing mode is applied [96,97]. However, some researchers also defined the separation efficiency differently, such as [98]:

(3) $$R\left(\%\right)=\frac{{\left(M_{\mathrm{water}}+M_{\mathrm{oil}}\right)}_{\mathrm{before}}-M_{\mathrm{water}\;\mathrm{after}}}{M_{\mathrm{oil}\;\mathrm{before}}}$$

where $M_{\mathrm{water}}$ and $M_{\mathrm{oil}}$ are the mass of the water and oil before and after the separation process, respectively. Zhang et al. [99] defined the separation efficiency as follows:

(4) $$R\left(\%\right)=100\left(\frac{V}{V_{0}}\right)$$

where V and $V_{0}$ are the volume of permeate and feed, respectively.

Coalescence is a demulsification process and it is an irreversible process. The destabilized oil droplets collide and combine into larger oil droplets and eventually form the oil slick floating on the water surface. The wettability and pore size of the nanofiber membrane are the main factors to determine the coalescence of the oil droplets [100,101,102]. The demulsification efficiency (α) can be employed to evaluate the ratio of residual emulsion in the permeate [103]:

(5) $$\alpha =\frac{10^{4}V_{s}}{\varphi_{d}V_{\mathrm{perm}}}$$

where $V_{s}$ is the total volume of the water phase layer and the oil/water emulsion layer in the permeate, $\varphi_{d}$ is the water content in the feed oi/water emulsion and $V_{\mathrm{perm}}$ is the volume of the permeate.

## 4. Thin-Film Composite Nanofiber Membrane for Oil/Water Separation

The crosslinked coating was used to improve the hydrophilicity in the electrospun nanofiber membranes and, thus, provide good antifouling characteristics. The coating materials must possess hydrophilic and highly water-permeable properties. The coating layer deposits on the electrospun nanofiber membrane surface must be sufficiently thin, but too thin a layer can lead to structural disintegration. Hence, optimization of the coating layer thickness must be investigated carefully. Although the added hydraulic resistance due to the coating layer can reduce the flux, the declination in the flux caused by the fouling of a membrane without the hydrophilic coating layer is much more severe, especially for the extended operation [104,105]. Yoon’s group successfully fabricated thin-film nanofibrous composite (TFNC) membranes by coating the electrospun PAN nanofibrous scaffolds with chitosan and PVA, which rejected the oil emulsion by at least 99%, respectively, in 24 and 190 h of operations [106,107].

Metal ions in the oily wastewater make the oil/water separation more challenging because the tiny sizes of the ions are difficult to retain by the membranes. The membrane surface charges used in the oil/water emulsion treatment significantly influence the demulsification and fouling [108,109]. Zhu et al. [110] fabricated the PVA-charged hydrogel nanofibrous membranes (CHNMs) by the electrospinning and crosslinking processes with glutaraldehyde and phytic acid. The surfaces of PVA CHNMs are negatively charged, which modified the stability in the negatively charged oil/water emulsion. The collision of the unstable oil droplets results in coalescence. The electrostatic repulsion between the negatively charged membrane surface and the negatively charged emulsion reduces the fouling. The separation between the oil and water is further enhanced when the PVA CHNMs are superhydrophilic and oleophobic. However, the investigation into various foulants, such as the natural organic matter, synthetic organic compounds produced during disinfection processes and soluble microbial products contained in the real oily wastewaters using the crosslinked nanofiber membranes, is scarcely reported.

## 5. Nanomaterials in Nanofiber Membrane for Oil/Water Separation

Different types of nanomaterials are used to modify the electrospun nanofiber membranes and, thus, improve the wettability properties and the antifouling characteristics in the new nanofiber membranes. For the oil/water separation application, the nanomaterials include silver (Ag) nanoparticles [101], gold (Au) nanoparticles [99], TiO_2_ nanoparticles [111], Fe_3_O_4_ nanopowder [112], silica nanoparticles [113], polydopamine nanoparticles [114], graphene oxide [115] and electrospun polystyrene nanofibers [116]. The modification process for the electrospun nanofiber membranes using the nanomaterials includes graft polymerization [117], coating [118], electrospinning [116], spraying [119] and incorporation of the nanoparticles in the base polymer solution before electrospinning [112].

Although most studies revealed the successful modified membranes can achieve high separation efficiencies, as shown in Table 3 and Table 4, for oil/water mixtures and oil/water emulsions, respectively, there are a few major concerns. The concerns include the reduction in the mechanical properties in the modified membranes [120,121]; adhesion of the nanomaterials [122]; applicability of the modified membranes in corrosive and harsh environment [123]; and health and safety of the use of chemicals [124].

## 6. Sustainable Development of Nanofiber Membrane for Oil/Water Separation

The nanomaterials were successfully applied in developing the nanofiber membranes for both oil/water mixture and oil/water emulsion treatments by upgrading the wettability to be either superhydrophilic or superhydrophobic. The superwetting nanofiber membranes can achieve separation efficiency of at least 90% and improve the flux simultaneously, as reported in the literatures. Despite that, the utilization of the chemicals is various and expensive. Bio-based nanomaterials derived from renewable materials, such as agricultural wastes, would be a better choice to increase the values of sustainability. Obaid et al. [115] reported that silica nanoparticles extracted from rice husk significantly improved the PSF electrospun nanofiber membrane fluxes for petroleum oil fractions/water separation. However, the separation efficiencies were not revealed in the study. Bioinspired silica nanoparticles have been synthesized from many biomass resources [135], such as rice husk [136,137], sugarcane bagasse [138], bamboo sticks and leaves [139], palm kernel shell [140], etc. Lignin-derived nanomaterials [141] are also potential precursors of nanofiber membranes for oily wastewater treatment in the future.

## 7. Conclusions and Remarks for Future Directions

Nanofiber membrane filtration is a promising technique to treat oily wastewater, either in the form of free-floating oil (>150 μm), dispersed and unstable oil/water emulsion (20–150 μm) or stable oil/water emulsion (<20 μm). The application of nanofiber membranes can compete with existing oil separation technologies in terms of economic, environmental and safety considerations. Even though many publications in scientific journals have been found in recent years, practical development on a commercial scale is still lacking. A few suggestions for future research are summarized as follows:

- Most of the oil/water emulsions tested in laboratories comprise two components. However, the real oily wastewaters discharged from numerous industries may contain abundant organic and inorganic compounds. These compounds may induce the nanofiber membranes to perform differently than the findings obtained from the binary mixtures. Some the organic compounds can swell the polymeric nanofibers and, eventually, may alter the nanofiber membrane properties. Investigation of using the real oily wastewaters in fouling and swelling could be an attractive topic in future research.
- Membrane surface modification to produce super wetting properties can improve the oil removal efficiencies. However, the preparation of the modified nanofiber membranes involves sophisticated procedures. Significant types of chemicals are expensive. Natural and sustainable resources with simple techniques for modified nanofiber membrane preparation are recommended in future studies.
- Most current oil/water separation studies use simple gravity-driven filtration systems and the membrane sizes are approximately 40–50 cm in diameter. To manage the large volumes of the oily wastewaters discharged from industries, a large-scale filtration system that can run for long-term operation is required.
- Modelling studies on oily wastewater and even oil/water separation using nanofiber membranes are hardly found in the literature. A vigorous model, which can accurately predict the nanofiber membrane performance, is required when scaling up the filtration system.

## Figures and Tables

**Table 1:** Types of polymeric materials used in different nanofiber production techniques.

Technique	Polymeric Material	Reference
Needle electrospinning	PVP, PAN, PVDF, PU, PEO, PLA, PCL, PES, Nylon 6, PSU, PVA, PET	[27,28,29,30,31,32,33,34,35,36]
Needleless electrospinning	PBS, PVA, EPS, PEO, PAN, PA, PCL, PLLA	[37,38,39,40,41,42,43]
Melt-blowing	PP, PU, PBT, PE, PS, PPS, Nylon 6, PLLA, TPVA	[44,45,46,47,48,49,50,51,52]
Melt-blending extrusion	PMMA, EVOH, PE, PET, PTT, PBT	[20,53,54,55,56]
Drawing	PAN, PCL, PEO, PET, PA, PVA, PVB, PMMA, HA,	[57,58,59]
Centrifugal force spinning	PVA, PLLA, Nylon 6, PAN, PHBV, PLGA, PS, PCL	[14,60,61,62,63,64]
Phase inversion	PLLA, PPTA	[65,66]
Template synthesis	PCL, PPy	[67,68]
Self-assembly	PA, PLLA, PAH, POM	[69,70,71]

PVP: Polyvinyl pyrrolidone; PAN: Polyacrylonitrile; PVDF: Polyvinylidene fluoride; PU: Polyurethane; PEO: Polyethylene oxide; PLA: Poly(lactic acid); PCL: Polycaprolactone; PES: Polyethersulfone; PSU: Polysulphone; PVA: Polyvinyl alcohol; PBS: Poly (butylene succinate), a bio-based polyester; EPS: Expanded Polystyrene; PLLA: Poly(L-lactide); PP: Polypropylene; PBT: Poly(butylene terephthalate); PE: Polyethylene; PS: Polystyrene; PPS: Poly(phenylene sulfide); TPVA: Thermoplastic polyvinyl alcohol; PMMA: Poly(methyl methacrylate); EVOH: Polyethylene-*co*-polyvinyl alcohol; PTT: Polytrimethylene terephthalate; PEO: Polyethylene oxide; PA: Polyamide; PVB: Polyvinyl butyral; HA: Hyaluronic acid; PHBV: Poly(3-hydroxybutyrate-*co*-3-hydroxyvalerate); PLGA: Poly-lactide-*co*-glycolide acid; PPTA: Poly(*p*-phenylene teraphthalamide); PPy: Polypyrrole; PAH: Poly(allylamine hydrochloride); POM: Polyoxometalate

**Table 2:** Comparison of different nanofiber production techniques.

Technique	Advantages	Disadvantages
Needle electrospinning	Scalable, feasible of fiber dimension control, fibers are long and continuous	Solvent recovery issues, low productivity, instable jetting, high voltage requirement
Needleless electrospinning	Scalable, feasible of fiber dimension control, fibers are long and continuous, high productivity	Solvent recovery issues, high voltage requirement
Melt-blowing	Scalable, feasible of fiber dimension control, fibers are long and continuous, high productivity, solvent recovery is not required	Number of suitable polymers is limited, high temperature requirement
Melt-blending extrusion	Scalable, feasible of fiber dimension control, fibers are long and continuous, high productivity, solvent recovery is not required	Number of suitable polymers is limited, high temperature requirement
Drawing	Simple process	Low scalability, incapable of fiber dimension control, discontinuous process
Centrifugal force spinning	Scalable, feasible of fiber dimension control, high voltage is not required	Require high temperature
Phase inversion	Simple equipment	Low scalability, incapable of fiber dimension control, limited to selective polymers
Template synthesis	Easy to modify the fiber diameter by using different size of template	Complex process
Self-assembly	Easy to obtain smaller nanofibers	Low scalability, incapable of fiber dimension control, complex process

**Table 3:** Application of nanomaterials in the nanofiber membranes for the oil/water mixture separation.

Base Polymer	Nanomaterials	Wettability	Oil/Water System	Oil Content in Water	Filtration Mode	J (L/m^2^ h)	R (%)	Findings	Reference
PVDF	P(MMA-*r*-FDMA)	Highly hydrophobic and superoleophilic WCA: 140 ± 5° OCA: <1° UWOCA: ~0° in ~0.6 s	Dodecane/water Dichloromethane/water	1:1 volume ratio	Gravity-driven	2500–3000 ^a^	-	Enhanced up to 7 times higher Young’s modulus; exhibited up to 17 times faster permeation of oil and organic solvent; highly stable and excellent fouling resistant during a 70 min continuous oil/water separation filtration; flux was 24 times higher than the pristine PVDF.	[125]
PI	SNPs (avg. size 7–40 nm)	Superhydrophobic and superoleophilic WCA: 155.75° OCA: <10°	Dichloromethane/water 1,2-dichloromethane/water Trichloromethane/water Carbon tetrachloride/water Bromobenzene/water	50%, *v*/*v*	Gravity-driven	>4400	98.81 99.36 99.55 98.07 98.40	Mimicked to a frogspawn structure; high resistance to damages due to high temperature (150 °C), acid/basic conditions and organic/inorganic solvents; the permeate flux greater than 4400 L/m^2^ h after 20 separation cycles.	[126]
PVDF	SNPs (avg, dia. 20 nm)	Superhydrophobic and superoleophilic WCA: 150.0 ± 1.5° OCA: 0°	Hexane/water Petroleum/water Vegetable oil/water Vacuum pump oil/water	1:1 volume ratio	Gravity-driven	1857 ± 101	99	Excellent multi-cycle performance and stable chemical resistance.	[113]
PI	SNPs (avg. size 7–40 nm)	Superhydrophobic and superoleophilic WCA: >154° OCA: ~0° in 30 s UWOCA: <20°	Dichloromethane/water Trichloromethane/waterDichloroethane/water Bromobenzene/water Carbon tetrachloride/water	1:1 volume ratio	Gravity-driven	4798	>99	A fluorine-free membrane dip-coated and in situ crosslinked with PBZ; superhydrophobicity was maintained after immersing in either acidic or alkaline aqueous solutions for 24 h; superhydrophobicity was maintained within 350 °C; high salt tolerance; good recyclability after 20 separation cycles; oil content in the permeate below 5 ppm	[124]
PVA	PTFE NPs (size ~200 nm)	Superhydrophobic and superoleophilic WCA: 155° SA: 5.1°	Chloroform/water	1:1 volume ratio	Gravity-driven	1215	-	Tensile strength was as high as 19.7 MPa compared with pristine PVA-PTFE at 7.5 MPa; superhydrophobicity was maintained after exposure to both acidic and alkaline solution for 2 h, and after 30 cycles of abrasion test.	[127]
PAN	Ag, Cu nanocluster	Superhydrophobic and superoleophilic WCA: 147.6–154.6° SA: 8.0°	Heavy oil mixture: Chloroform/water Light oil mixtures: Motor oil/water Diesel/water Toluene/water	1:1 volume ratio	Gravity-driven	-	>99.40 ^b^>98.50 ^c^	The PAN-Cu-Sh-120 membrane exhibited WCA greater than 150° after immersed in different NaCl concentration solutions for up to 7 days; no change in weight before and after ultrasonic treatment which indicated the adhesion strength of copper nanocluster to PAN was strong; elongation at break decreased from 26.07 to 11.79% after electroless deposition Cu.	[98]
PP	PDA/APTES	Superhydrophilic and underwater superoleophobic WCA: 0° UWOCA: >150°	Petroleum ether/water Toluene/water	50:50 volume ratio	Gravity-driven	186,477.5 202,935.5	>99 ^d^	PDA created nano-scale roughness on the fiber; APTES improved the adhesion or interactions between the PDA coatings and PP; breaking elongation reduced from 52% to 36% when the basis weight of PP membrane increased.	[44]
PP	TiO_2_	Hydrophobic and superlipophilic WCA: 130–140° OCA: 0°	Kerosene/water Hexane/water Petroleum ether/water Toluene/water	1:1 volume ratio	Gravity-driven	14,789–15,410	95–98	TiO_2_ enhanced the thermostability of PP; thermal decomposition temperature was proportional to the content of TiO_2_ which the temperatures were 180–230 °C; remained stable after 6 h ultraviolet irradiation; retained the oil/water separation capability even after 100 repeated test.	[128]
PP	TP/APTES	Superhydrophilic and underwater superoleophobic WCA: 0° in few seconds UWOCA: >150°	n-hexane/water cyclohexane/water petroleum ether/water kerosene/water colza oil/water	1:1 volume ratio	Gravity-driven	~110,000 ~99,000 ~90,000 113,000 49,000	>99.1	The *R* was maintained at 99.8% after 30 cycles of separation; UWOCA kept at above 153° after immersed in ultrasonic water for a long time; UWOCA remained above 150° after immersed into various inorganic salt solutions and solutions pH 2 to pH10 for 24 h; TP/APTEST coating decomposed in the solution pH 12 and greater.	[129]

^a^ Continuous filtration flux. ^b^ Separation efficiency of heavy oil/water mixture calculated based on Equation (3). ^c^ Separation efficiency of light oil/water mixture calculated based on Equation (3). ^d^ Separation efficiency was calculated based on Equation (4). PVDF: Polyvinylidene fluoride; P(MMA-*r*-FDMA): Poly(methyl methacrylate-*random*-perfluorodecyl methacrylate); PI: Polyimide; PBZ: Polybenzoxazine; PVA: Polyvinyl alcohol; PTFE: Polytetrafluoroethylene; TP: Tea polyphenols; WCA: Water contact angle; OCA: Oil contact angle; UWOCA: Underwater oil contact angle; SA: Sliding angle; SNPs: Silica nanoparticles; NPs: Nanoparticles; APTES: (3-Aminopropyl)triethoxysilane.

**Table 4:** Application of nanomaterials in the nanofiber membranes for oil/water emulsion separation.

Base Polymer	Nanomaterials	Wettability	Oil/Water System	Oil Content in Water	Filtration Mode	J (L/m^2^ h)	R (%)	Findings	Reference
PAN	Single-walled CNTs (OD: <2 nm, L: 5–30 μm)	Switchable hydrophobic and hydrophilic	Petroleum ether/water	1:9 volume ratio	Vacuum driven at −0.07 MPa	~55,000	99.96	Hydrophobic CNT side and hydrophilic PAN side.	[96]
PVDF	SNPs (dia. 30 nm, 50 nm, 200 nm, 1 μm)	Hydrophobic and oleophilic WCA: 135° OCA: 0° in 2 s UWOCA: 87°	Octane/water Hexadecane/water Diesel oil/water Rapeseed oil/water	500–2000 mg/L	Dead-end, 0–10 kPa	-	97.95 98.60 92.70 90.80	Exhibited excellent performances in oil-water separation for the flow velocities below 1.98 m/min; surface roughness and pores increased the probability of droplets capture by interception and collision.	[130]
N6	SNPs	Superhydrophilic and underwater oleophobic WCA: 0° in 1 s UWOCA: 116°	Machine/water + SDS	250–1000 mg/L	Dead-end stirred cell filtration, 4 psi	4814 ^a^	>98.80	SNPs increased the surface roughness from 193 to 285 nm; incorporation of SNPs enhanced the tensile strength to 22.48 MPa due to the integrated network structure; strong interaction between the N6 nanofiber and PVAc coat maintained the stability after permeation with acidic and alkaline solutions for 3 h.	[131]
PVDF	PDA and TiO_2_	Superhydrophilic and underwater superoleophobic WAC: 0° in 1–34 s UWOCA: 158.6°	Diesel oil/water *n*-hexadecane/water 1,3,5-trimethylbenzene/water Petroleum ether/water	1:100 volume ratio + 0.2 mg/ml SDS	Vacuum filtration, ΔP at 0.09 MPa	785	99.52 99.34 99.13 98.86	The modified membrane exhibited excellent stability under acidic, salty and physical stress; PDA disintegrated in a strongly alkaline environment; superhydrophlicity maintained and no loss of NPs even after strong shear flow at 30°C for 30 days.	[132]
PAN	Electrospun PS	Hydrophobic WCA: 113–126°	Hexane/water	1 mL hexane in 99 mL deionized water, 0.1 wt% SDS	Gravity-driven	209–1841 ^b^ 227–430 ^c^	-	Emulsion flux of J-ENMs was 1.7 times higher than that of single layer PAN NF; PS concentrations affected emulsion fluxes.	[116]
PAN	Ag, ZnO	Superhydrophilic and underwater superoleophobic WCA: 0° in 0.6 s UWOCA: 154.4°	Soybean oil/water	1% soybean oil mixed with 20 mg/L cationic dye or anionic dye	Gravity-driven	619	>99.7	Micro/nano sized hierarchical structure greatly increased the roughness; strong resistance to different pH solutions, organic solvents and salt solutions for 24 h with WCA and UWOCA maintained;	[119]
PAN	Au	Superhydrophobic and underwater superoleophilic WCA: ~155.5° OCA: ~0° UOWCA: ~158°	Chloroform/water	6 ml chloroform in 0.54 g Tween 80 and 54 mL water	Gravity-driven	-	97.8 ^d^	Separation efficiency maintained at 85% after 16 cycles of separation;	[99]
PAN	TiO_2_	Superhydrophilic and superoleophobic OCA: 166–162°	Petroleum ether/water Bump oil/water Soybean oil/water	1:1000 weight ratio with 0.1 mg/mL Tween 80 in water 1:99 weight ratio without surfactant	Gravity-driven, 0.01 bar	600–2000	99	Emulsion property such as viscosity affected the separation efficiency; no obvious decline of permeation; robust recyclability; soybean emulsion flux decreased quickly with time because the oil drop size was smaller.	[111]
PAN	PDA	Superhydrophilic and underwater superoleophobic WCA: 0° in 0.12 s UWOCA: 165 ± 1°	Toluene/water	3.0 ml in 0.03 g SLS and 297 mL deionized water	Gravity-driven	11,666 ± 978 ^e^	99.9	Micro/nano-spehres formed in the PAN-PDAc; permeability of PAN-PDAc NF was about 2.7 times of the pristine PAN; initial permeability of PAN-PDAc was 23.3% higher than PAN; the permeability after 2 h in PAN-PDAc was 174.8% higher than PAN.	[114]
PP	TA/DA/PEI	Superhydrophilic and underwater uperoleophobic WCA: 0° in 1 s UWOCA: >154°	1,2-dichloroethane/water Toluene/water *n*-hexane/water Cyclohexane/water Petroleum ether/water	10 mL in 990 mL deionized water with 20 mg Tween-80	Gravity-driven	- 463 ± 30 489 ± 24 509 ± 35 513 ± 32	99.8	Mussel-inspired hydrophilic structure; tannin-inspired coating used to improve the adhesion; oil droplets form filter cake and block the pores on the surface; *R* was greater than 95% even after 10 cycles for 1,2-dichloroethane/water; some TA/DA/PEI particles detached in alkaline solutions at pH12 and pH14; more stable for acidic, weak alkaline and organic solvents.	[133]
PET	Electrospun PVDF NF	Hydrophobic and lipophilic WCA: 130° OCA: 0° UWOCA: 65.7°	Hexadecane/water Octane/water Diesel/water Rapeseed oil/water	Concentration of oils ranged from 500 to 2000 mg/L	Dead-end filtration	-	~99 ~98 ~95 ~92	*R* increased from 73.0% to 99.5% when the number of electrospun PVDF NF layer increased from 1 to 4; *R* decreased to 95.8% for 5 layers of electrospun PVDF NF; *R* for the highly viscous oil (rapeseed oil) was slightly low due to the difficulty of the oil collided and coalesced.	[134]

^a^ Permeability measured in unit L/m^2^ h bar. ^b^ Pure water flux. ^c^ Emulsion flux. ^d^ Separation efficiency calculated based on Equation (4). ^e^ Permeability in unit L/m^2^ h bar. PAN: Polyacrylonitrile; PVDF: Polyvinylidene fluoride; N6: Nylon 6; PVAc: Polyvinyl acetate; PDA: Polydopamine; PS: Polystyrene; DA: Dopamine; PEI: Polyethyleneimine; PET: Polyester. WCA: Water contact angle; OCA: Oil contact angle; UWOCA: Underwater oil contact angle; UOWCA: Under oil water contact angle; SNPs: Silica nanoparticles; NPs: Nanoparticles; CNTs: Carbon nanotubes; SDS: Sodium dodecyl sulfate; SLS: Sodium laurylsulfonate; NF: Nanofiber.

## Data Availability

Not applicable.
